# Explaining uncertainty in women's fertility preferences

**DOI:** 10.1016/j.heliyon.2024.e27610

**Published:** 2024-03-11

**Authors:** Amke M.G. van Tintelen, Gert Stulp

**Affiliations:** a University of Groningen, University Medical Center Groningen, Department of General Practice & Elderly Care Medicine, PO Box 196, 9700 AD, Groningen, the Netherlands; b Midwifery Academy Amsterdam Groningen, InHolland, Groningen, the Netherlands; c Amsterdam UMC location Vrije Universiteit Amsterdam, Midwifery Science, De Boelelaan 1117, Amsterdam, the Netherlands; d University of Groningen, Department of Sociology & Inter-University Center for Social Science Theory and Methodology, Grote Rozenstraat 31, 9712 TS, Groningen, the Netherlands

**Keywords:** Fertility preferences, Uncertainty, The Netherlands, Partner agreement, Family size

## Abstract

People's fertility preferences are often considered an important determinant of fertility. What is often neglected in studies of preferred fertility, is the uncertainty that people may have about their preferences. In this study, using data on Dutch women through the Longitudinal Internet studies for the Social Sciences (collected early 2018), we examined women's fertility preferences and asked detailed questions about the certainty of these preferences. We also examined whether women agreed with their partner on preferred family size, and to what extent partner (dis)agreement shaped uncertainty. We show that Dutch women expressed much uncertainty about their fertility preferences, with only one-third feeling strongly about their preferences. Uncertainty strongly increased when women preferred higher numbers of children, whereas already having children reduced it. Women who wanted no children were most certain about their preference. Higher preferred family sizes also led to more disagreement with the partner about these preferences, and greater partner disagreement, in turn, led to more uncertainty. These findings imply that people are more likely to downgrade their fertility preferences than to increase them, as women are more certain about their preferences for lower numbers of children and are more open to family sizes below than above their preferred choice. Partner disagreement is often resolved by not having (more) children, lowering realised fertility. Hence, these findings provide another explanation for why many people have fewer children than desired.

## Highlights

• Dutch women expressed much uncertainty about their fertility preferences.
• Mothers and women with a partner were more certain about their preferred family size.
• Certainty of fertility preferences decreased with each additional preferred child.
• Couples more often agree on smaller than larger preferred family sizes.

## 1 Introduction

In many contemporary Western populations, a gap exists between how many children people prefer, which is approximately two, and how many children people have, on average fewer than two [1,2]. Major factors contributing to this gap are the rising ages at childbearing, adverse economic circumstances, and medical infertility [[1], [2], [3], [4]]. Such research typically assumes people's preferences are fixed or static, whereas these preferences are susceptible to life circumstances [5,6]. Less prominence has been given to the idea that fertility preferences themselves are uncertain and may contribute to this gap [7]. In this study, we examine the strength of or uncertainty about people's expressed preferences and the (dis)agreement couples may have about preferred family size. We argue that both of these factors are more likely to lead to family sizes smaller than initially preferred rather than larger family sizes.

Previous research shows that people are uncertain about their fertility preferences [[8], [9], [10]], which is described as “the extent to which a person knows how she feels or what she wants” [11]. People also vary in the strength of their preferences [7,12,13]; some may feel strongly about a particular family size whereas others are not attached to a particular number but have a range of acceptable family sizes in mind [14]. If people are less certain about their preferences for larger family sizes than for smaller family sizes or if people are more accepting of family sizes below than above ideally preferred [7], then it is expected that realised fertility is often lower than preferred fertility.

Partner disagreement about preferred family size may be another reason why realised fertility is often lower than personal preferences [13,15]. Fertility decisions are generally joint decisions; both partners' fertility desires matter in fertility outcomes [13,[16], [17], [18]]. In the absence of strong assortative mating for fertility preferences, partners are likely to hold different preferences [15]. If one partner prefers not to have (more) children, couples are less likely to become parents [16] or pursue additional births [18,19]. Thus, ideal fertility tends to be downgraded when partners disagree [7].

The current study is an investigation into the certainty and strength of expressed fertility preferences in a sample of Dutch women. This study advances previous research by the detailed way of asking about women's fertility preference and their (dis)agreement with their partner about these preferences. Furthermore, we explicitly examine how certainty and agreement vary by preferred family size to investigate whether more uncertainty and disagreement occur at larger preferred family sizes. Last, we identify which personal characteristics are most strongly associated with women's certainty about their fertility preferences. Results are discussed in light of recent debates on the value of measuring fertility preferences. Findings help explain part of the gap between preferred and realised fertility.

## 2 Background

### 2.1 Stability of fertility preferences

Fertility preferences or desires reflect the number of children people would like to have, irrespective of possible constraints in achieving these preferences [13]. Many people in Western countries have fewer children than they desire [1,2], which is referred to as the fertility gap. This gap is often explained by factors that restrict the realisation of desires or intentions, such as medical infertility and competing preferences [1,2]. However, the fertility gap can also occur due to the manner in which fertility preferences are typically measured. Indeed, fertility preference measures have received their fair share of critique [13,20]. For instance, ambiguity and inconsistencies exist in the different forms of measuring fertility preferences [20,21]. Moreover, fertility preferences should not be seen as static but as a moving target that changes over the life course [4,6,22] depending on life circumstances, including experiences with earlier births [23]. Bhrolcháin and Beaujouan [20] even argue that fertility preferences are constructed at the time of the survey based on available information at that time rather than being some fixed number retrieved from memory (see also [24]).

The criticism of the measurement of fertility preferences is corroborated by studies on the stability of fertility preferences, which show that fertility preferences are anything but a stable number that persists throughout the life course [8,[25], [26], [27], [28]]. For example, about one in three women changed their preferred family size within a period of four years, of which half decreased their preference [28]. Other studies suggest that when preferences are revised, they are revised downwards to smaller ideal family sizes [8,26]. The stability of fertility preferences further depends on the number of children women prefer; women who expected larger families were more likely to revise their expectations downwards than respondents who expected to have smaller families [26,28]. Women who preferred to have no children or two children were found to have the most stable preferences [25,26].

Researchers have attempted to explain the instability of fertility preferences through the life-span theory of control. This theory suggests that people rely on two control strategies for achieving goals: primary and secondary control [29]. Primary control reflects an extrinsic process that comprises behaviour to change the environment so it aligns with an individual's desires. Secondary control, in contrast, reflects an intrinsic process of adapting desires which are unlikely to be achieved to desires that are more likely to be achieved. As individuals might have little power to change the environment when it comes to fertility outcomes, and thus primary control might be low, secondary control might help to explain the instability of fertility preferences. Secondary control could thus result in adapting fertility preferences when the realisation of these preferences becomes unlikely [6]. This form of control, in which the desired family size is adjusted, helps to deal with failure to achieve goals [29]. Given that fecundability declines with age [30], the life-span theory would suggest that women revise their fertility preferences downwards over time to make the realisation of these preferences more likely. This, in turn, makes preferences seem unstable.

### 2.2 Uncertainty in and strength of fertility preferences

Another explanation as to why fertility preferences appear unstable over time is that changes in preferences express uncertainty about these preferences rather than an adjustment of preferences [31]. The instability of fertility preferences in previous studies can thus also be seen as an expression of uncertainty about those preferences [11]. Studies on the desired family size have often overlooked uncertainty and instead focused on a single reported desired family size or even disregarded expressions of uncertainty in analyses [25,26,28]. Nonetheless, previous studies that take uncertainty into account showed that a substantial number of women are uncertain about their desired family size [8,10]. For example, Morgan [10] found that about 10% of the women are uncertain about their intention to (not) have a child, as indicated by the answer “don't know”. Additionally, women who expressed the intention to have a child often expressed uncertainty about this intention; 40% of the women thought they might change their mind and could decide not to have another child. Women who did not intend to have a child were more certain and less often indicated they might change their mind (17%). In studies by Berrington [8,9], women were considered to be uncertain about their family size preferences if they indicated that they did not know whether they wanted any (more) children or that they wanted children but did not know how many. Of the women in their early twenties, about 10% were found to be uncertain about their intended number of children [8,9].

In addition to uncertainty about whether to have children and how many, there is also a related concept of the strength of preferences. For example, even when people express their preferred family sizes there can be variation in the willingness of people to deviate from this preference; some may have one ideal number in mind whereas others have a range of equally preferred options [7,12]. In one of the few studies focusing on the strength of fertility preferences, Hin and colleagues [7] extended the measurement of fertility preferences by including alternative preferences. Respondents were asked to indicate to which extent they preferred one preferred family size over another one, resulting in the 'relative strength of preferences'. Hin and colleagues [7] found that a substantial number of men and women had no strong preference for their first preferred family size over their second preferred family size; only about 20% of the respondents felt strongly about the first preference. Although many respondents had no strong preferences, the preferences were found to be internally consistent [7]: people who initially preferred fewer children were more open to lower-than-ideal alternatives, whereas people who preferred more children more often increased the preferred number of children when asked about their alternative preference.

#### 2.2.1 Determinants of the uncertainty and strength of fertility preferences

An important determinant of fertility preferences and certainty about those preferences is people's experience with parenthood. Being a parent reduces uncertainty about what it means to raise children and as such parents may have a better idea about their preferred number of children [32]. In contrast, childless women have not experienced parenthood, making their preferences more abstract and possibly less certain. Childless women were indeed found to be more uncertain about their fertility preferences than mothers [8,31]. In a sample of British women, uncertainty about the intended number of children ranged from 2% among mothers in their late thirties to almost 30% among childless women in their early thirties [8]. While Berrington [8] only considered women who answered “don't know” to questions about their fertility intention to be uncertain, Bhrolcháin and Beaujouan [31] adopted a broader definition of uncertainty, also including women that answered “probably yes”, or “probably not” when asked whether they intended to have a(nother) child. Despite the differences in operationalisation, Bhrolcháin and Beaujouan [31] also found lower levels of uncertainty among mothers (24%) than among childless women (42%). An exception may be women who desire to be childfree. Childfree lifestyles are increasingly accepted [33], but women who choose such a lifestyle still face social pressure and stigma [34]. ‘Coming out’ as childfree may then be an expression of the strength of the preference.

Age also contributes to uncertainty about fertility preferences, but differently so for women with and without children. For women without children, highest levels of uncertainty were found for women aged 30 to 34 at 29% [8,31]. This was 16% for mothers of the same age who had one child [8]. The normative age deadline for childbearing could explain why women in their late thirties are more certain than women in their early thirties. In a Dutch sample, two-thirds of the respondents indicated there is an age after which women are too old to have a child [35]. Due to such norms, uncertainty could turn into certainty with respect to intending any (more) children for women in their late thirties.

Another determinant of how strongly people feel about their fertility preferences is having a partner. Women with a partner may be more certain because the intention to conceive might also reflect the intention to conceive with a particular partner [36]. Women without a partner also face the uncertainty of finding a partner (who potentially will have different preferences) that may be reflected in their fertility preferences.

### 2.3 Partner agreement on fertility preferences

Another source underlying the instability or uncertainty of one's preferences may be the partner disagreement on such preferences. Generally, among childless couples, there is substantial agreement on the preference to have at least one child [8,37,38]. Couples that have one child also tend to agree to have at least one more child [8,37]. Conversely, couples that have two or more children most often indicate agreement on not intending to have more children [8,37].

Although these studies show that most couples agree on whether they want to have children or not, some studies show considerable disagreement about the number of children. Manea and Fucik [39], for instance, showed that 80% of couples agreed on whether to have children, whereas 64% of couples agreed on the ideal number of children. A study by Schytt [38] among childless couples yielded similar results; 70% of the couples agreed on whether to become a parent or not, but less than half (48%) of the couples that wanted children agreed on the desired number of children. In the majority of the couples that wanted children but disagreed on the desired number of children, the women preferred to have more children than their partners [38].

Partner disagreement is another factor that can explain the fertility gap between ideal and realised fertility [13,15]. Couples are more likely to have children when both partners in the couple want to [18,37,40]. If lower preferences prevail in couples with conflicting fertility preferences, realised fertility will be lower than preferred fertility [15].

### 2.4 The current study

Here we expand on this research by examining the uncertainty and strength of preferences in a detailed way and relating the strength of preference to the preferred family size. We examine the extent to which parenthood status, partnership status, and age change peoples’ certainty about their preferences. We further advance previous research by examining the little-studied topic of partner agreement on preferred family sizes. This research can contribute to explaining the gap between preferred and realised fertility: if people feel stronger about having smaller families than having larger families, those who prefer to have more children might have fewer children than desired compared to those who prefer smaller family sizes, resulting in a gap between preferred and realised fertility. Moreover, if agreement on fertility preferences promotes childbearing, realised fertility might be lower than the preferred family size when partners disagree.

## 3 Methods

### 3.1 Participants

In this paper, we use data from the LISS (Longitudinal Internet studies for the Social Sciences) panel administered by Centerdata (Tilburg University, The Netherlands). The LISS panel is a representative sample of Dutch individuals who participate in monthly Internet surveys and is based on a true probability sample of households drawn from the population register by Statistics Netherlands (CBS). Much effort was put into ensuring a representative sample and high response rates. The resulting representativeness of the LISS panel was similar to those of traditional surveys based on probability sampling [41,42]. Refreshment samples substantially corrected initial selection biases, and further refreshment samples were planned for attrition biases [43].

The LISS panel allows researchers to conduct their survey within the panel; thus, we added a study named the Social Networks and Fertility Survey that focused on social influences on fertility desires and outcomes. For this survey, all women in the LISS panel between the ages of 18 and 40 (N = 1332) were invited to participate between February 20 and March 27, 2018. In total, the survey was completed by 758 women. Women who responded did not substantially differ from those who did not on a range of demographic variables. Ethical approval for this particular study was obtained through the ethical committee of sociology at the University of Groningen (ECS-170920). See Stulp [44,45] for further details on the study. Data can be accessed through: https://www.lissdata.nl.

### 3.2 Procedure

The first part of the survey concerned questions about fertility and partnerships. The second part of the questionnaire involved detailed questions on the social networks of these women [44,[46], [47], [48]], which will not be included in this study. The phrasing and operationalisation of the questions and concepts were reviewed by a team of family sociologists, demographers, and survey specialists. The focus of this study is on the following items:

*Preferred number of children*: "How many children would you like to have? This is including the X children you already have."; options: 0–10, More than 10, I don't know.

*Strength of preference*: "You indicated in the previous question that you would like to have X children/X more children than you currently have. We would like to know how strong this preference is." See Table 1 for answer categories and how we recoded them. This question was not asked to women who responded with “I don't know” on the preferred number of children (N = 77).

**Table 1:** All possible answer categories to the question concerning the strength of the fertility preference (sample size in brackets). The columns logistic- and multinomial regression explain how categories are collapsed for analyses.

Answer categories (n)	Logistic regression	Multinomial regression
I'd rather not have more or fewer than [previously mentioned #] children (211)	Certain	Rather not more/fewer
More children would be fine, but I'd rather not have fewer (45)	Uncertain	More is fine
Fewer children would be fine, but I'd rather not have more (74)	Uncertain	Fewer is fine
I really would like to become mother (again), but the number of children is not important (105)	Uncertain	Number is not important
I don't care much about the number of children; one child more or fewer is fine (109)	Uncertain	Number is not important
I don't care much whether I will have (more) children (29)	Uncertain	Uncertain
I don't really know (108)	Uncertain	Uncertain

*Relationship status*: “Do you currently have a partner? By a partner, we mean somebody that you are in a relationship with for over three months. Husbands are also considered partners.”; options: (1) Yes, (2) No.

*Agreement with partner about the preferred number of children*: "Which statement best reflects the situation between you and your partner?" See Table 2 for answer categories and how we recoded them. This question was only asked to women who indicated in a previous question that they had talked to their partner about having children.

**Table 2:** All possible answer categories to the question concerning the agreement with the partner on preferred fertility (sample size in brackets). The columns logistic- and multinomial regression explain how categories are collapsed for analyses.

Answer categories (n)	Logistic regression	Multinomial regression
We both like to have the same number of children (195)	Agreement	Agreement
We both would not like to have (more) children (112)	Agreement	Agreement
We both would like to have (more) children, but we haven't discussed the preferred number (97)	Disagreement	Number not discussed
I would like more children, but my partner doesn't (28)	Disagreement	Partner wants fewer
My partner wants fewer children than I do (29)	Disagreement	Partner wants fewer
I don't want more children, but my partner does (8)	Disagreement	Partner wants more
My partner wants more children than I do (30)	Disagreement	Partner wants more
I don't know (23)	Disagreement	Don't know
Did not discuss preference with partner (32)^a^	Disagreement	Don't know

a In a different question, women indicated whether they had talked to their partner about having children. We incorporated those who responded that they did not discuss this with their partner in our measures of agreement.

### 3.3 Analysis

Our two main measures of interest, the strength of preference and the agreement with partner on fertility preferences are categorical variables which is why we opted for logistic- and multinomial logistic regressions. We examined the extent to which preferred family size, age, having children, and having a partner were associated with measures of the (un)certainty and strength of the preferred number of children. In examining the strength of the preference, we excluded women who did not know how many they preferred (N = 77). In a subsequent model, we also examined whether partner agreement improved the model.

We further examined partner (dis)agreement about fertility preferences. Respondents who indicated they had no partner or who indicated they did not know how many children they preferred were excluded from the analysis (leaving N = 510 for analyses). In these models, we included the variables age, parenthood, and the preferred number of children.

Of the variables of interest, some categories contained only a few cases (see Table 1, Table 2). Moreover, there were very few or even no cases for particular combinations of categorical variables (see supplementary materials), which hinders the reliability of statistical estimates. This is why we collapsed our variables of interest into fewer categories (see Table 1, Table 2). For all models, this resulted in each cell having at least 10 cases. The only exception was the model in which partner agreement was used to predict the strength of preferences (with cell counts of 5 and 8). We ran a robustness check where we further reduced the number of categories in partner agreement to 3 categories and re-estimated the model (see supplementary material).

To assess the magnitude of the effects of the variables in predicting the outcome, we use average adjusted predicted probabilities estimated through the R-package marginaleffects [49]. These are calculated by estimating the change in probability (controlling for other variables in the models) for each respondent in the dataset after a value for one variable is changed (e.g., increasing age by one year for each respondent or changing relationship status from not having a partner to having a partner). We then calculate the average of these changes across all respondents. These measures are particularly suited for multinomial logistic regression in which many comparisons between categories are made.

We ran 1000 bootstraps for each model, which meant that the model was re-estimated on a dataset that was sampled from the original dataset (with replacement). For each of the 1000 bootstraps we calculated the average predicted probabilities for each of the variables in the model. We present the mean of the 1000 bootstrap estimates as our estimate of the change in probability, and the 95% confidence interval is determined by the 2.5% and 97.5% percentile from the 1000 bootstrap estimates. We also calculate a p-value by assessing in how many bootstraps predicted probabilities are above and below 0. As an example, in estimating the effect of having children on the strength of the fertility preference, we observed a mean effect of 0.20 with a 95% interval ranging from 0.11 to 0.30 and a p-value of 0.000 on the probability of responding with “rather not more/fewer children”. This meant that, compared to not having children, having children increases the probability across respondents to answer “rather not more/fewer children” by 20%-points [95% CI: 11–30%]. In 0 of the 1000 bootstrap samples an effect below zero was observed.

Estimates for all logistic- and multinomial logistic regression models can be found in the supplementary materials. For all models, we report likelihood ratio tests to assess whether a variable significantly impacted model performance and to assess whether a full model significantly improved an intercept-only model. As a different measure of model fit, we calculated the proportion of correct classifications. The percentage of correct in-sample classifications is an overestimate of the ‘true’ percentage when having to predict novel, unseen cases. To get a better estimate of this out-of-sample predictive accuracy, we used 5-fold cross-validation [50]. We partitioned the data into 5 ‘folds’ (random partitions of the data), 4 of which were used to train the models and the fifth (test data) was used to get an estimate for the out-of-sample predictive accuracy. We used each fold as test-data once, and we averaged the percentage of correct classifications across these 5 folds. Here we only report on the average adjusted probabilities for the variables and out-of-sample accuracy for the full models that we believe are the most relevant and robust measures of effect size. We mostly focus on variables that led to a 5%-point change or larger in the outcome. We only report the average change and not the confidence intervals and p-value, which can be found in Fig. 3, Fig. 4 and the supplementary tables and figures.

## 4 Results

### 4.1 Descriptive statistics

**Fig. 1:**
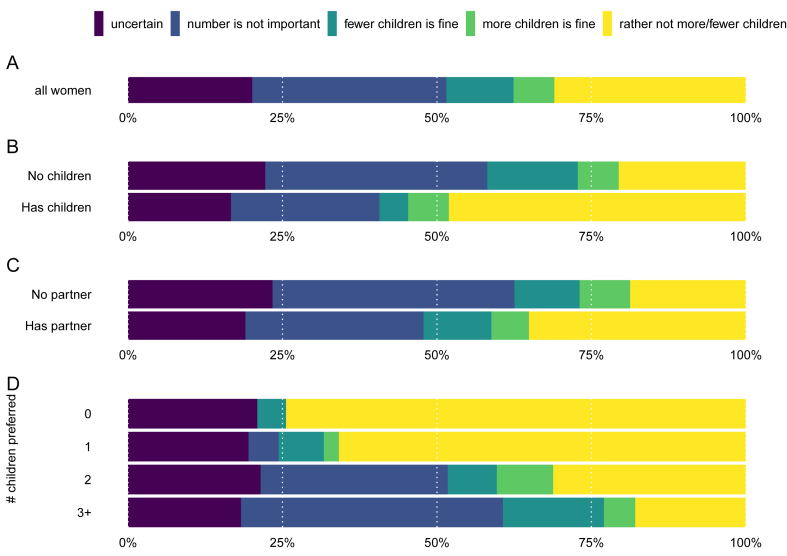
Percentage of women (N = 681) with varying degrees of certainty about their preferred number of children (A), depending on motherhood status (B), partnership status (C), and the preferred number of children (D).

**Fig. 2:**
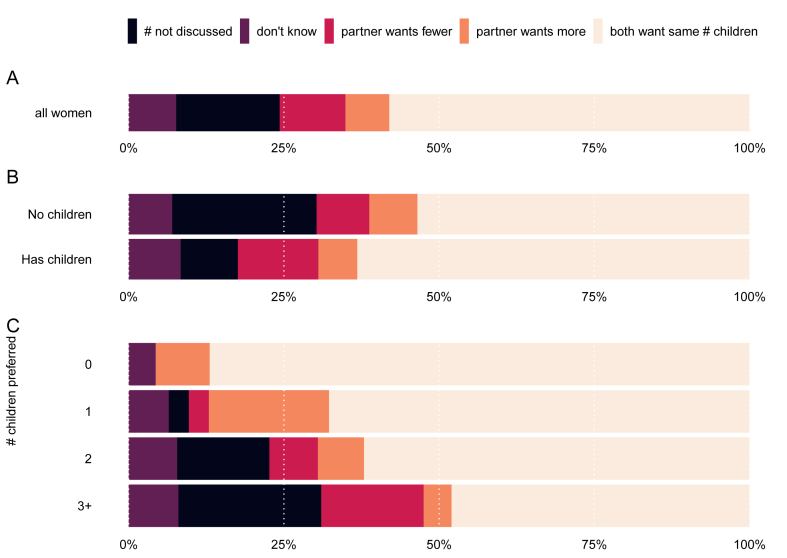
Percentage of women (N = 510) with varying degrees of agreement with her partner on the preferred number of children (A), depending on motherhood status (B), and the preferred number of children (C).

Women were, on average, 29 years old (SD = 6.5; age was unknown for one woman). There were 273 mothers and 485 women without children in our sample, and 554 women had a partner. The average number of preferred children was 2.3 (SD = 1.0). See Table 1, Table 2, Table 3 and Fig. 1 ABCD and Fig. 2 ABC for further descriptive statistics.

**Table 3:** Descriptive statistics.

	*n* (*%*)
**Preferred number of children**^1^**(N = 758)**	
0	43 (6%)
1	41 (5%)
2	340 (45%)
3 or more	257 (34%)
I don't know	77 (10%)
**Motherhood status (N = 758)**	
No children	485 (64%)
Mothers	273 (36%)
**Partnership status (N = 758)**	
No partner	204 (27%)
Partner	554 (73%)
**Strength of preference (N = 681)**	
Rather not more/fewer	211 (31%)
More is fine	45 (7%)
Fewer is fine	74 (11%)
Number is not important	214 (31%)
Uncertain	137 (20%)
**Agreement with partner (N = 554)**	
Agreement	307 (55%)
Partner wants more	38 (7%)
Partner wants fewer	57 (10%)
# Not discussed	97 (18%)
Don't know	55 (10%)

### 4.2 Strength of preference

**Fig. 3:**
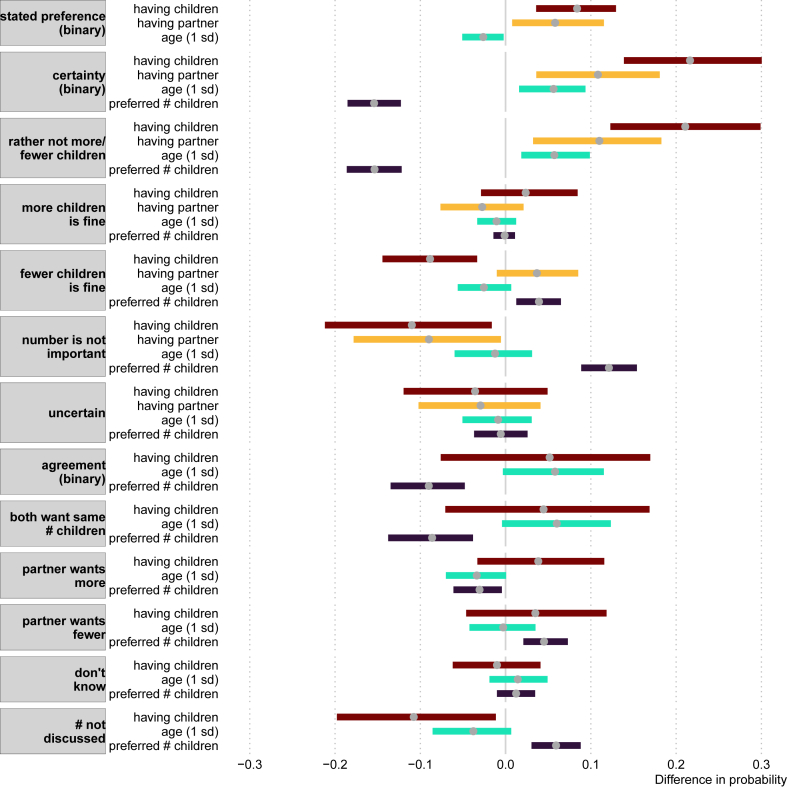
Average difference in adjusted predictions on several outcomes (in grey) for the variables having children (reference: no children), having a partner (reference: no partner), age, and the preferred number of children (difference in probability with a change of respectively 1 SD and 1 unit). The average (grey dot) and the 95% confidence interval (width of bar) is based on 1000 bootstrap samples.

Seventy-seven women reported “I don't know” rather than stating a preferred number of children. Having children, having a partner, and being younger increased the probability of stating a preference (Fig. 3). Having children increased the probability of stating a preference by 8%-points, and having a partner by 6%-points. The effect of age was smallest: a standard deviation increase in age led to a 3%-points reduction in stating a preference. The full model could not outperform an intercept-only model both with an out-of-sample accuracy of 90%.

**Fig. 4:**
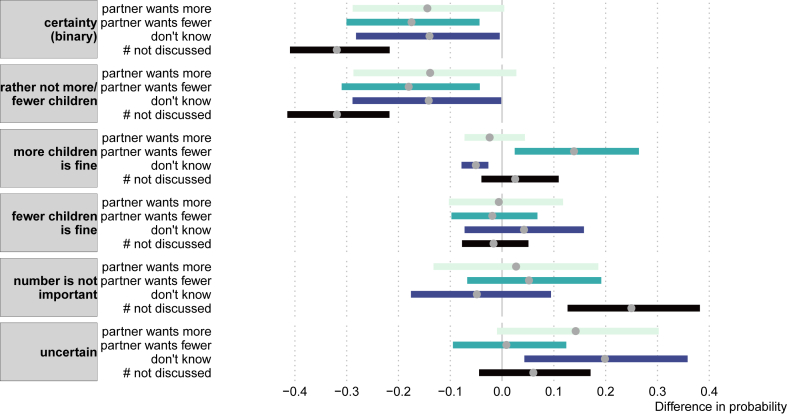
Average difference in adjusted predictions on several outcomes (in grey) for different categories of partner agreement (reference: partner agreement). The average (grey dot) and the 95% confidence interval (width of bar) is based on 1000 bootstrap samples.

Only women who indicated a preferred family size (N = 681) reported their strength of preference (Table 3; Fig. 1A). About one-third of these women indicated they felt strongly about their preference: they would rather not have more or fewer children than the preferred number (31%). In contrast, 20% of women were uncertain, and 31% indicated the number of children was not important. Women were furthermore more likely to indicate “fewer children is fine, but not more” (11%) than “more children is fine, but not fewer” (7%). After collapsing these categories into certain or uncertain (see Table 1; Fig. 3), a logistic regression revealed that the preferred number of children had a strong effect on being certain: an increase in the preferred number of children of 1 led to a decrease in certainty of 15%-points. Certainty was increased by having children (22%-points), having a partner (11%-points), and being older (6%-points for SD increase in age). The out-of-sample accuracy was 76%, an improvement over the 69% from the intercept-only model.

We then ran a multinominal logistic regression using the strength of preference outcome with five categories (see Table 1; Fig. 2ABC; Fig. 3). These results corroborated the findings of the logistic regression of being certain (i.e., reporting “rather not have more or fewer children”). There was further evidence that the number of preferred children decreased the strength of preference, as it increased the chance of saying “the number is not important” by 12%-points, and of saying “fewer children is fine” by 4%-points. In contrast, having children decreased these chances by 11%- and 9%-points respectively. Also having a partner decreased the chance of saying that the number is not important by 9%-points. Age was not strongly associated with any other category than being certain. The out-of-sample accuracy for this model was 47%, which is a substantial increase over the 31% of the null model.

Because the preference to be childfree may be qualitatively different from answers of one and up because of the social stigma and pressure childfree individuals face [7,34,51], we also ran a model comparing those who preferred to be childfree to those who preferred to have children. Preferring to be childfree was associated with a 57%-points increase in being certain about this preference compared to individuals who preferred to have children (see supplementary materials).

### 4.3 Agreement with partner

Another source of uncertainty in fertility preferences may come from partner disagreement on the preferred number of children. Of the respondents with a partner who indicated a family size preference (N = 510), more than half indicated agreement with their partner on the preferred number of children (Fig. 2; 58%). A smaller share of couples agreed to want children but did not discuss the number (17%). Having a partner who preferred fewer children (11%) was more common than having a partner who preferred to have more children (7%). The remaining women did not know or had not discussed the issue with their partner (8%).

We first examined to what extent partner (dis)agreement can be explained by whether women have children, their age, and their preferred family size (Fig. 3). A logistic regression showed that an increase in the number of preferred children led to a 9%-point decrease in agreement. Partner agreement was not particularly well-predicted by these three variables, as the out-of-sample predictive accuracy for the full model was 59% and for the null model 58%.

A multinomial regression using five distinct categories of partner agreement as outcome (see Table 2; Fig. 3) revealed that the preferred number of children had the strongest effect and decreased agreement by 9%-points. The preferred number of children furthermore increased the probability of the “partner wanting fewer” (by 5%-points) and the probability of not having discussed the number (by 6%-points). Having children also decreased the probability of not having discussed the preferred number (by 11%-points). Age further increased the probability of agreement by 6%-points. Overall model fit was again poor, as the out-of-sample predictive accuracy of the full model was lower than that of the null model.

In our final analyses, we added partner agreement as a predictor of the strength of preferences. Through a logistic regression on certainty (Table 1; Fig. 4), we found that each category of partner disagreement relative to agreement reduced the probability of being certain. The largest effect, a reduction of 32%-points in certainty was found for couples that wanted children but that had not discussed the number of preferred children compared to couples that agreed. The other categories of disagreement (relative to agreement) also led to substantive reductions in certainty by about 15%-points. The out-of-sample predictive accuracy of the model was 76% which was much higher than the 65% of the null model.

A multinomial regression showed nearly identical results for the category “rather not more/fewer children” (i.e., being certain; Fig. 4). Other categories of the strength of preference were less well predicted by agreement, although when couples had not discussed the number of children, they were much more likely to say that the number is not important (an increase in 25%-points; compared to couple agreement). Also, when women reported to not know about the preferred number of the partner, they were 20%-points more likely to report uncertainty about their fertility preference. Finally, when the partner wanted fewer children, there was a 14%-points increase in reporting “more children is fine”. The out-of-sample predictive accuracy of the model improved was 48% which was a substantial improvement over the 35% of the null model.

## 5 Conclusion and discussion

Dutch women expressed much uncertainty about their fertility preferences. First, one in ten women did not know their preferred family size. Second, when women reported a preferred family size, they expressed uncertainty about this preference, with only one-third feeling strongly about their preference and rather not having fewer or more children. Thus, in the current study, where we explicitly ask about uncertainty in preferences, we observe that even a larger percentage of women express uncertainty than in previous studies (e.g., 10–20% in [8,10]).

The strongest determinant of the uncertainty in women's fertility preferences was preferred family size; certainty decreased with each additional preferred child. Women who did not want to have children were most certain (only ∼25% expressed uncertainty in their preferred family size), whereas women who wanted three or more children were least certain (∼80% expressed uncertainty). An increase in the preferred number of children also increased the likelihood of reporting that fewer children than preferred would be fine, whereas a decrease in the number of children did not lead to more reporting of more children being fine. The preference to be childfree was very strong: women who preferred to be childfree were 57%-points more likely to be certain about their preference than women who wanted children.

These results corroborate earlier findings that people are more certain about lower than higher fertility preferences [7] and that uncertainty was higher among women who preferred to have children than women who preferred to remain childless [10]. The finding that women who do not want children are most certain may be explained by the barriers these women face when expressing the desire to remain childless. Women who express the desire to remain childless receive higher social scrutiny and stigma and are more often asked to explain their preference than those who want to be a parent [7,34,51]. Thus, women with a childfree preference may have thought about their preferences more. When they are comfortable in expressing their preference despite the social stigma and scrutiny, this is likely because they feel strongly about it.

Mothers were more certain about their preferred number of children than women without children. While half of the mothers in the sample were certain about their preferred family size, only about one in five childless women was certain, which mirrors findings by Berrington [8] and Bhrolcháin and Beaujouan [31]. The reason mothers are more certain is likely due to their experiences with parenthood, which allows them better insights into what having additional children would entail thereby strengthening fertility preferences [23,32]. Women with a partner were more certain about their preferred family size, although (not) having a partner was less important for certainty than both motherhood status and preferred family size. Not having a partner increases uncertainty about future fertility trajectories that may be reflected in fertility preferences.

This study was unique in asking in a detailed way about partner agreement on fertility preference. In the current study, 59% of the women indicated agreement with their partner, which falls between earlier estimates of 48% and 64% in studies questioning both partners [38,52]. It does mean that more than four in ten women disagreed with their partner about the preferred family size, did not discuss the desired number of children, or did not know their partners’ preferences. Also, in partner agreement, the preferred family size was the most important determinant: over 90% of women who prefer to remain childless indicate they agree with their partner on the preferred number of children, compared to about half of the women who prefer three or more children.

Mothers agreed on the number of children with their partner more often than childless women, though the differences were small. Previous studies found an opposite association: parents more often disagreed on the intention to have children than childless couples [8,53,54]. Two possible explanations for these differences in results lie in the measurement of agreement. First, the measure used in our study allowed for more answers, such as ‘want children, did not discuss number’, and ‘I don't know’. Previous studies did not allow for these answers or, as in the study by Gibbs and Moreau [53], ‘don't know’ responses were coded as the intention to have more children. Hence, the possibility of more precisely indicating the level of agreement might have affected the results. Second, we assessed agreement based on perceptions from the respondents about their partner rather than from the partners themselves. An important disadvantage of this approach is that the level of agreement with a partner about fertility desires is generally overestimated [55].

Partner (dis)agreement is also an independent determinant of women's certainty about their preferred family size: greater disagreement led to more uncertainty. A possible explanation is that women who perceive agreement on the preferred number of children are more certain as their fertility preferences are affirmed by their partner. Moreover, among women who initially disagreed on the preferred number of children but eventually reached a compromise, the process of reaching a compromise may have increased certainty as the preferred number of children is more thoroughly thought through.

These findings can be important for understanding the fertility gap, widely observed in low-fertility Western countries [1], where men and women have fewer children than desired. As women are more certain about their preferences for lower numbers of children and are more open to family sizes below than above their preferred choice, it is not surprising that realised fertility is often lower than preferred fertility. Similarly, partner disagreement is often resolved by not having (more) children [16,18,19], lowering realised fertility. Altogether, our results underscore the limitation of using a single, stated preference as a robust measure of people's reproductive decision-making [20,24,56].

A clear limitation of our study is that we focus on only Dutch women. When it comes to fertility, the Netherlands is rather unique [57]: the total fertility rate of the Netherlands has been relatively high without active fertility policies. Moreover, despite the Netherlands being a highly secular society, cultural norms of fathers as primary breadwinners and mothers as homekeepers prevail, also reflected by the high rates of part-time work for women and an aversion for formal childcare [57]. Such normative paths for men and women may reduce feelings of uncertainty. At the same time, there is widespread tolerance for a childfree lifestyle [33], that both may increase or decrease certainty as it presents a viable alternative lifecourse. It is unclear to what extent our findings generalise to other European countries where more gender-equal norms prevail (e.g., Scandinavian countries) or to countries where particular lifestyles (e.g., childfree lifestyle) are less broadly accepted (e.g., eastern European countries [58]).

Like many other Western countries, contraception is widely available and accessible in the Netherlands, and this is also true for medically assisted reproduction. This facilitates people in achieving their desired family size, although the major postponement of first births results in lower fertility and an increase in the fertility gap. Our results cannot straightforwardly speak to populations with an unmet need for contraception [59], where substantial fractions of people have more children than desired.

The current study is also limited in that it provides little insights into the reasons and feelings behind individuals' fertility preferences and the uncertainty of these preferences. Future research will benefit from a qualitative approach to explore possible causes of uncertainty (see e.g., [60]). People's narratives of the future may be key in understanding their uncertainty [61]. Future quantitative research could focus on additional contributors to strength of preferences to get a better understanding of reproductive decision-making. For example, information on people's goals and ambitions (i.e., competing alternatives; [62]) would likely help explain who are more certain in their desired family sizes. Another factor that can affect the strength of preferences for future births is the gender composition of children. For example, Mishra and Parasnis [63] show that in a context in which son preferences prevail, parents are more likely to intend to have a third child if they have two daughters. In European countries, particularly among countries with high gender equality, there is a mixed-gender preference [64]. Parents are more likely to have a third child if they have two same-sex children [64].

These findings may have several societal implications. First, the substantial uncertainty people have about their preferences as well as how levels of certainty vary with preferred family size suggest that preferences are a poor barometer for future fertility. Second, the gap between preferred and realised fertility is often interpreted as an unmet need for children that should be addressed by family policies. Our results suggest that this gap is an overestimate of this unmet need (see also [65]), as for many people preferences are not particularly strong, and preferences are weaker for higher preferred numbers of children. This is not to argue that we should think about the fertility gap lightly: for individuals with strong preferences to have children, not having children can come at severe costs of wellbeing. Family policies should facilitate people in achieving their fertility desires (also in light of contemporary low fertility rates), although the success of such policies is often limited [66]. For couples with a strong pro-natal preference, it is important to learn (for example through pre-conception counselling) what would be the best timing to have reasonable chances of fulfilling their preferences [67].

## Ethical statement

Participants of the LISS panel followed a double informed consent procedure. Ethical approval for this particular study within the LISS panel was obtained through the ethical committee of sociology from the University of Groningen (ECS-170920).

## Data availability statement

Data from this study can be accessed here: https://www.dataarchive.lissdata.nl/study_units/view/1377. The data are freely available but registration is required. Materials to reproduce the findings in the current manuscript in addition to supplementary materials can be found here: https://doi.org/10.34894/PLXSE6.

## Funding information

The LISS panel data were collected by Centerdata (Tilburg University, The Netherlands) through its MESS project funded by the Netherlands Organization for Scientific Research. This work was further supported by the Netherlands Organization for Scientific Research, VENI grant number 451-15-034 to GS.

## CRediT authorship contribution statement

**Amke M.G. van Tintelen:** Writing – review & editing, Writing – original draft, Formal analysis, Conceptualization. **Gert Stulp:** Writing – review & editing, Writing – original draft, Visualization, Supervision, Project administration, Methodology, Investigation, Funding acquisition, Formal analysis, Data curation, Conceptualization.

## Declaration of competing interest

The authors declare that they have no known competing financial interests or personal relationships that could have appeared to influence the work reported in this paper.
